# Differences in Symptoms among Black and White Patients with ME/CFS

**DOI:** 10.3390/jcm11226708

**Published:** 2022-11-12

**Authors:** Leonard A. Jason, Chelsea Torres

**Affiliations:** Center for Community Research, DePaul University, 990 W. Fullerton Ave., Chicago, IL 60614, USA

**Keywords:** ethnicity, fatigue, gender, myalgic encephalomyelitis/chronic fatigue syndrome

## Abstract

Study samples of patients with myalgic encephalomyelitis/chronic fatigue syndrome (ME/CFS) have primarily involved White subjects, so the literature on ethnic differences is sparse. The current study identified a sample of 19 Black patients diagnosed with ME/CFS and compared them with White patients with ME/CFS, as well as with healthy controls. The studies used a similar psychometrically sound assessment tool to assess symptoms in all subjects. Findings indicated there were significant differences between patients with ME/CFS versus controls, but few differences between patients who identified as Black or White. The results suggest there might be few symptom differences between patients with ME/CFS in these two ethnic groups. The implications of these findings are discussed.

## 1. Introduction

Research suggests that ethnic groups may differ in their experience of fatigue. For example, in a community-based sample of adults, Song et al. (1999) found fatigue severity scores for Black and Latino individuals were significantly higher than for White individuals. In addition, Black women had significantly higher rates of fatigue when compared to Black men, and older Black men had significantly higher rates of fatigue than younger Black men [1]. These differential ethnic findings for fatigue might in part due to the fact that non-White participants experience higher social strain, perceived discrimination, and depression [2].

Potential differences between Black and White samples among those with six or more months of fatigue have rarely been thoroughly examined. In an exception to this, Taneja et al. [3] compared the prevalence and pain severity of symptoms among a sample of Black and White participants with six or more months of fatigue, from a community-based sample. Significant differences in symptom prevalence were found in the areas of disturbed sleep and reproductive activity. Black patients also experienced more pain due to symptoms related to orthostatic intolerance.

In the literature, patients with myalgic encephalomyelitis/chronic fatigue syndrome (ME/CFS) have been described as being mostly white, upper-class women, but these samples were primarily recruited from clinical and hospital-based settings [4,5]. Relatively few studies have examined patients with ME/CFS among groups of color. A few community-based studies have found that non-white patients with ME/CFS experience more severe symptoms than White individuals [6]. For example, in a community-based study, Jason et al. [7] found that people of color with ME/CFS experienced more severe sore throat pain, more severe post-exertional malaise, more severe unrefreshing sleep, poorer general health, and less optimism regarding their illness. Although a small sample size precluded examining individual ethnic groups, these findings suggest people of color with ME/CFS, including those identifying as Black and Latino, may be more severely ill and experience poorer general health when compared with White samples.

Broadly, Black individuals have been documented in the literature to have a greater prevalence of a range of health conditions when compared with other racial or ethnic groups [1], and this might be due to the historical and contemporary social and economic disparities faced by the Black community. While many health conditions occur at a higher rate in Black patients, it is unclear whether there are symptom differences between Black and White patients with ME/CFS. The current study examined this question among Black and White patients with ME/CFS.

## 2. Methods and Materials

Our study identified 19 Black patients that were part of several ME/CFS studies conducted at DePaul University. All studies employed a comparable assessment tool called the DePaul Symptom Questionnaire (DSQ). A large aggregate data set of 2308 patients with ME/CFS was collected from the US, Europe, and Japan [8]. This large data set was collected through various methods from tertiary clinics to internet-based surveys. Another data set involved a prospective study of college students who developed ME/CFS (*n* = 55) after being exposed to infectious mononucleosis [9]. A final data set involved 42 patients with ME/CFS from a pediatric community-based prevalence study [10].

Within these samples, we identified 19 Black patients with ME/CFS, and they were matched with 19 White patients with ME/CFS based on age and sex. We also included 19 Black and 19 White healthy controls, also matched on age and sex. Educational levels among the groups were not significantly different. ME/CFS diagnosis was based on different criteria, as some studies involved a physical examination by a physician and others relied on self-reporting of ME/CFS.

The DSQ is a structured, self-report instrument with 54 major symptoms that relate to ME/CFS criteria [11]. For each symptom item, respondents are asked to separately rate the frequency and severity over the last six months on a 5-point Likert scale. Symptoms are contained in seven major domains: Neurocognitive, Neuroendocrine, Pain, Orthostatic, Sleep, Post-Exertional Malaise (PEM), and Immune. The DSQ has demonstrated high test–retest reliability among patients and controls [12], shown strong internal consistency [8], and yielded valid, clinically useful results [13]. Moreover, the DSQ has been used to accurately differentiate those with ME/CFS from those with other chronic illnesses [14,15].

An analysis of variance was used to identify differences among the four groups, and if a significant difference was found, then Bonferroni planned comparisons were conducted between the four groups.

## 3. Results

Table 1 provides the means and standard deviations for DSQ symptoms across the four groups (Black individuals with ME/CFS, White individuals with ME/CFS, Black control participants, and White control participants). Among the seven major domains, the Black patients with ME/CFS evidenced directionally higher and more troubling scores for 27 symptoms and four domains (Neurocognitive, Neuroendocrine, Pain, and Orthostatic) whereas the White patients with ME/CFS evidenced directionally higher symptoms for 21 symptoms and 3 domains (Sleep, PEM, and Immune). However, the two ME/CFS groups significantly differed on only one item, with Black patients with ME/CFS having higher chest pain than White patients with ME/CFS.

Unsurprisingly, the patient ME/CFS groups had directionally higher symptom scores on all items in comparison to the healthy controls. Black patients with ME/CFS had significantly higher scores than Black controls on 19 items, including five of the seven domains (Sleep, PEM, Neurocognitive, Pain, and Orthostatic). However, when Black patients with ME/CFS were compared with the White controls, there were 27 significant differences, including six of the seven domains (all except Immune). Therefore, there were eight more significant differences among symptom items and one more domain area when the Black patients with ME/CFS were compared with the White controls than when compared with the Black controls

When the White patients with ME/CFS were compared with the Black controls, there were significant differences for only 13 items, and three domains (Sleep, PEM, and Orthostatic). However, when the White patients with ME/CFS were compared with the White controls, there were significant differences for 21 symptoms and six of the seven domains (all but Immune). In other words, there were again eight more significant symptoms and three more domain differences when the White patients with ME/CFS were compared with the White controls than with the Black controls.

When comparing the two control groups, the Black controls had directionally worse scores on 40 items and six domains, whereas the White controls had directionally worse scores on only nine items and one domain.

## 4. Discussion

The study’s main finding was that there were few significant differences between symptomatologies of Black patients and White patients with ME/CFS. There were six more items and one more domain where the group of Black patients with ME/CFS had directionally worse scores than the White patients with ME/CFS. However, there was only one item that significantly differentiated the two patient groups, and that was higher severity of chest pain for Black patients with ME/CFS. Overall, these findings do point to Black patients with ME/CFS being more directionally impacted by symptoms than White patients with ME/CFS, but overall scores were relatively similar.

In contrast, Black controls when compared with White controls had directionally worse scores on 40 of the 49 symptom items and all except one of the domains. The Black controls as a comparison group were more directionally impaired than the White controls when contrasted with patients with ME/CFS. This finding supports prior work indicating more impairment among Black subjects in community samples when compared with White counterparts [16].

It remains unclear whether there are differences between Black and White ME/CFS samples regarding prognosis and treatment outcomes. Large samples as in the current study are needed to begin answering such questions, but most existing studies have used relatively small samples which were primarily White. Because people of color have greater disparities in health care accessibility, it is likely that current studies have not included a significant portion of the ME/CFS population [17,18]. Other factors that might hinder identification of patients include a lack of awareness of the illness and a preference for managing symptoms without medical intervention [19]. It is of importance for future research to overcome these barriers, so we might obtain a better understanding of ethnic differences within the ME/CFS patient population.

A major limitation encountered in this study was the sample sizes, even though relatively large datasets were available. In addition, these data did not include certain biological variables (e.g., immune markers), which might have resulted in different outcomes. We only had matching data on age, sex, and education, so we were not able to match the samples on other key sociodemographic variables such as BMI. Finally, the datasets were collected using different methods, with some confirming ME/CFS diagnosis by a medical healthcare worker and others relying on the patients’ self-report, some involving recruitment from tertiary care settings and others relying on the internet. Research involving community-based methodology and relying on more accessible means of participant recruitment (i.e., not requiring established access to a healthcare professional or internet connectivity) is imperative.

In conclusion, although only one significant result did emerge when the two patient groups were compared, it does appear that among the control groups, the Black controls had more directionally troublesome symptoms than the White controls. In contrast, for the ME/CFS patient groups, fewer differences were found. It is certainly possible that in the general population, those who are Black have more symptoms, as has been found in other general population studies (Song et al., 2002), but given the severe health status of those with ME/CFS (Lim et al., 2020), Black and White populations both have comparable symptoms. However, firm conclusions on this issue will need to be confirmed by future research with larger samples.

## Figures and Tables

**Table 1:** ME/CFS versus controls; White versus Black.

	ME/CFS		Control	
	Black	White	Black	White
	*n* = 19	*n* = 19	*n* = 19	*n* = 19
	M (SD)	M (SD)	M (SD)	M (SD)
**Sleep**	48.42 (21.35) ^a,b^	50.1 (22.8) ^c,d^	28.82 (16.4) ^a,c^	25.07 (18.83) ^b,d^
Unrefreshing sleep	76.97 (17.81) ^a,b^	78.95 (21.67) ^c,d^	45.39 (21.33) ^a,c^	43.42 (29.57) ^b,d^
Needing to nap	47.92 (31.87) ^a^	57.24 (30.71) ^b,c^	27.08 (19.76) ^b^	17.11 (28.93) ^a,c^
Problems falling asleep	47.37 (32.96)	48.61 (35.33)	27.78 (27.64)	26.39 (25.69)
Problems staying asleep	31.94 (29.77)	37.5 (36.32)	17.11 (24.72)	22.37 (25.2)
Waking up early	27.94 (28.14)	26.97 (30.41)	21.71 (24.59)	15.13 (21.07)
**PEM**	46.86 (24.06) ^a,b^	52.41 (19.22) ^c,d^	22.81 (18.94) ^a,c^	14.8 (17.95) ^b,d^
Heavy feeling	37.5 (33.21)	44.74 (29.26) ^a,b^	18.42 (17.86) ^a^	15.13 (20.66) ^b^
Soreness	41.45 (33.09) ^a^	49.34 (23.38) ^b,c^	21.71 (24.95) ^c^	9.87 (17.47) ^a,b^
Mental fatigue	41.45 (34.12) ^a^	48.03 (28.34) ^b,c^	20.39 (26.75) ^c^	13.82 (24.61) ^a,b^
Tired little exercise	52.63 (32.7) ^a,b^	61.18 (23.9) ^c,d^	27.63 (31.89) ^a,c^	9.87 (18.44) ^b,d^
Drained	38.81 (32.78) ^a,b^	45.39 (27.7) ^c,d^	14.47 (17.31) ^a,c^	8.55 (16.69) ^b,d^
Fatigue	69.08 (18.8) ^a,b^	65.79 (21.99) ^c,d^	34.21 (21.99) ^a,c^	31.58 (28.98) ^b,d^
**Neurocognitive**	47.36 (23.85) ^a,b^	39.62 (20.09) ^c^	26.46 (20.6) ^a^	17.32 (18.34) ^b,c^
Memory problems	53.29 (31.41) ^a^	36.84 (24.82)	32.89 (30.96)	19.08 (31.56) ^a^
Attention difficulties	63.16 (33.46) ^a,b^	53.95 (29.18)	34.87 (31.62) ^a^	30.92 (30.15) ^b^
Trouble with words	43.42 (29.57)	43.42 (25.81)	30.26 (27.74)	24.34 (29.89)
Difficulty understanding	46.05 (33.86) ^a^	37.5 (26.35)	28.29 (27.9)	17.76 (27.42) ^a^
Unable to focus	57.24 (34.69) ^a,b^	44.08 (31.83) ^c^	30.92 (27.44) ^a^	9.21 (21.18) ^b,c^
Slowness of thought	43.42 (33.94) ^a,b^	35.53 (24.74)	19.74 (21.78) ^a^	13.16 (23.74) ^b^
Absent-mindedness	50.0 (32.27) ^a^	36.84 (27.15)	27.63 (25.88)	15.13 (18.9) ^a^
Muscle twitches	21.71 (25.29)	32.24 (26.78)	17.76 (24.76)	11.84 (17.42)
Sensitivity to smells	45.83 (29.08) ^a,b^	36.18 (26.32) ^c^	15.79 (21.81) ^a^	14.47 (21.35) ^b,c^
**Immune**	21.05 (17.87)	21.93 (12.72)	13.16 (12.21)	10.96 (11.13)
Sore throat	26.97 (24.03)	30.26 (18.78)	21.71 (16.05)	17.76 (16.83)
Tender/sore lymph nodes	18.42 (21.4)	17.11 (18.73)	11.84 (22.23)	8.55 (11.82)
Fever	15.97 (19.56)	18.42 (15.79)	5.92 (11.31)	6.58 (14.05)
**Neuroendocrine**	27.55 (20.05) ^a^	25.66 (15.87) ^b^	16.12 (15.94)	10.2 (10.68) ^a,b^
Sweating hands	10.53 (18.29)	23.03 (25.77) ^a^	11.84 (20.19)	3.29 (8.17) ^a^
High temperature	28.95 (28.28)	28.29 (22.38)	13.82 (21.61)	14.47 (18.76)
Low temperature	14.47 (27.09)	13.16 (16.91)	5.92 (15.79)	1.34 (3.94)
Weight changes	30.92 (31.83)	24.34 (30.75)	20.39 (27.7)	13.82 (27.61)
Chills	30.92 (28.68) ^a^	29.61 (16.78)	19.74 (21.78) ^a^	11.18 (16.61)
Feeling hot or cold	48.03 (32.88) ^a,b^	34.87 (29.34)	22.37 (28.44) ^a^	17.76 (16.61) ^b^
Night sweats	26.97 (29.83)	23.68 (32.25)	17.76 (22.56)	5.92 (14.65)
No appetite	29.61 (32.33)	28.29 (30.0)	17.11 (22.9)	13.82 (17.13)
**Pain**	44.08 (20.94) ^a,b^	40.26 (21.62) ^c^	25.23 (14.75) ^a^	20.35 (19.75) ^b,c^
Muscle pain	57.89 (30.68) ^a^	53.95 (30.63) ^b^	33.33 (19.65)	26.32 (28.53) ^a,b^
Headaches	53.29 (31.96) ^a^	55.26 (27.74) ^b^	40.13 (22.66)	28.47 (28.05) ^a,b^
Joint pain	37.5 (38.64)	38.16 (34.73)	16.45 (15.62)	18.06 (30.38)
Eye pain	30.26 (34.69) ^a^	15.15 (20.23)	14.47 (24.74)	6.58 (10.51) ^a^
Abdomen/stomach pain	41.45 (29.48)	38.82 (31.15)	22.92 (18.32)	23.03 (24.74)
**Orthostatic**	33.33 (20.08) ^a,b^	28.95 (20.46) ^c,d^	9.54 (13.11) ^a,c^	9.82 (11.53) ^b,d^
Nausea	31.25 (25.81) ^a^	34.21 (27.9) ^b^	9.21 (16.58) ^a,b^	20.39 (26.75)
Chest pain	42.36 (29.44) ^a,b,c^	23.03 (23.3) ^a^	5.56 (15.59) ^b^	6.58 (14.05) ^c^
Feeling unsteady	26.97 (28.34)	29.61 (28.63)	9.21 (20.35)	11.18 (20.37)
Shortness of breath	44.44 (29.46) ^a,b^	36.84 (32.93) ^c^	16.45 (17.7) ^a^	10.53 (15.17) ^b,c^
Dizziness or fainting	36.18 (28.23) ^a,b^	35.53 (21.35) ^c,d^	13.82 (23.53) ^a,c^	8.33 (16.61) ^b,d^
Irregular heartbeats	13.82 (19.94)	14.47 (20.52)	3.29 (11.67)	1.32 (5.74)

Note: Similar letters across rows indicate significant differences. *p* < 0.05.

## Data Availability

Data can be obtained by contacting the first author.
